# Left Vocal Cord Paralysis, Lung Function and Exercise Capacity in Young Adults Born Extremely Preterm With a History of Neonatal Patent Ductus Arteriosus Surgery—A National Cohort Study

**DOI:** 10.3389/fped.2021.780045

**Published:** 2022-01-03

**Authors:** Mette Engan, Merete S. Engeset, Lorentz Sandvik, Ole C. O. Gamlemshaug, Ingvild Ø. Engesæter, Knut Øymar, Maria Vollsæter, Ola D. Røksund, Karl Ove Hufthammer, Thomas Halvorsen, Hege H. Clemm

**Affiliations:** ^1^Department of Clinical Science, University of Bergen, Bergen, Norway; ^2^Department of Pediatric and Adolescent Medicine, Haukeland University Hospital, Bergen, Norway; ^3^Faculty of Health and Social Sciences, Western Norway University of Applied Sciences, Bergen, Norway; ^4^Department of Otolaryngology and Head and Neck Surgery, Haukeland University Hospital, Bergen, Norway; ^5^Department of Pediatric and Adolescent Medicine, Stavanger University Hospital, Stavanger, Norway; ^6^Centre for Clinical Research, Haukeland University Hospital, Bergen, Norway; ^7^Department of Sports Medicine, Norwegian School of Sport Sciences, Oslo, Norway

**Keywords:** infant: extremely premature, infant: extremely low birth weight, vocal cord paralysis, cohort studies, patent ductus arteriosus, ligation, bronchopulmonary dysplasia, exercise test

## Abstract

**Background:** Left vocal cord paralysis (LVCP) is a known complication of patent ductus arteriosus (PDA) surgery in extremely preterm (EP) born neonates; however, consequences of LVCP beyond the first year of life are insufficiently described. Both voice problems and breathing difficulties during physical activity could be expected with an impaired laryngeal inlet. More knowledge may improve the follow-up of EP-born subjects who underwent PDA surgery and prevent confusion between LVCP and other diagnoses.

**Objectives:** Examine the prevalence of LVCP in a nationwide cohort of adults born EP with a history of PDA surgery, and compare symptoms, lung function, and exercise capacity between groups with and without LVCP, and vs. controls born EP and at term.

**Methods:** Adults born EP (<28 weeks' gestation or birth weight <1,000 g) in Norway during 1999–2000 who underwent neonatal PDA surgery and controls born EP and at term were invited to complete questionnaires mapping voice-and respiratory symptoms, and to perform spirometry and maximal treadmill exercise testing. In the PDA-surgery group, exercise tests were performed with a laryngoscope positioned to evaluate laryngeal function.

**Results:** Thirty out of 48 (63%) eligible PDA-surgery subjects were examined at mean (standard deviation) age 19.4 (0.8) years, sixteen (53%) had LVCP. LVCP was associated with self-reported voice symptoms and laryngeal obstruction during exercise, not with lung function or peak oxygen consumption (VO_2_peak). In the PDA-surgery group, forced expiratory volume in 1 second z-score (z-FEV_1_) was reduced compared to EP-born controls (*n* = 30) and term-born controls (*n* = 36); mean (95% confidence interval) z-FEV_1_ was −1.8 (−2.3, −1.2), −0.7 (−1.1, −0.3) and −0.3 (−0.5, −0.0), respectively. For VO_2_peak, corresponding figures were 37.5 (34.9, 40.2), 38.1 (35.1, 41.1), and 43.6 (41.0, 46.5) ml/kg/min, respectively.

**Conclusions:** LVCP was common in EP-born young adults who had undergone neonatal PDA surgery. Within the PDA-surgery group, LVCP was associated with self-reported voice symptoms and laryngeal obstruction during exercise, however we did not find an association with lung function or exercise capacity. Overall, the PDA-surgery group had reduced lung function compared to EP-born and term-born controls, whereas exercise capacity was similarly reduced for both the PDA-surgery and EP-born control groups when compared to term-born controls.

## Introduction

Extreme preterm (EP) birth is associated with a number of perinatal complications causing short- and long-term morbidity (1, 2). A patent ductus arteriosus (PDA) is diagnosed in ~40% of very low birth weight (<1,500 g) neonates and in 66% of EP-born neonates (3, 4). This shunt may give rise to cardiovascular dysfunction with pulmonary overcirculation and systemic hypoperfusion associated with worsening of lung disease, prolonged mechanical ventilation, increased risk of pulmonary hemorrhage, necrotizing enterocolitis, and intraventricular hemorrhage (5). Treatment options for PDA include a conservative symptomatic approach, pharmacological intervention, or surgical closure, the latter option usually representing a last resort (3, 6).

The left recurrent laryngeal nerve loops around the aorta in close proximity to the ductus arteriosus and left-sided vocal cord paralysis (LVCP) caused by iatrogenic nerve injury is a recognized complication of PDA surgery (7). Affected neonates may present with a weak cry, stridor, hoarseness, aspiration, and feeding problems (8, 9). Symptoms may be vague, and the condition can therefore pass unrecognized unless particularly examined for (10). Studies on EP-born neonates that report routine post-operative laryngoscopy have found incidences of LVCP ranging from 11 to 67% (7, 11).

Long-term consequences of LVCP in EP-born subjects beyond the first year of life are insufficiently described. A previous small study on EP-born adults who underwent neonatal PDA surgery discussed the possibility that LVCP occurring in the neonatal period may contribute to the long-term development of airway obstruction in this population (12), however, further research is needed on this topic. Moreover, both voice problems and breathing difficulties during physical activity could be expected with an impaired laryngeal inlet (13). More knowledge on long-term consequences of LVCP in the preterm population may prevent confusion between LVCP and other diagnoses with similar symptoms such as asthma or exercise-induced laryngeal obstruction (EILO).

As a group, premature infants who undergo PDA surgery may be particularly vulnerable to long-term health problems. PDA surgery has been associated with both bronchopulmonary dysplasia (BPD) and poor neurological outcomes (5, 8). Furthermore, several studies have found EP-born subjects to have reduced exercise capacity compared to term-born peers (14). We hypothesized that EP-born adults with a neonatal history of PDA surgery are at increased risk of impaired pulmonary and cardiorespiratory function, and that LVCP is associated with poorer outcomes.

We aimed to investigate the prevalence of LVCP in young adults born EP who underwent open PDA surgery in Norway during 1999–2000. Secondly, we aimed to compare self-reported voice and breathing symptoms, lung function, exercise capacity, and laryngeal obstruction during exercise between subjects with and without LVCP. Finally, we aimed to compare the lung function and exercise capacity in those who underwent PDA surgery with those of comparable EP-born controls and term-born controls.

## Methods

### Subjects and Study Design

This was a nationwide observational follow-up study of all individuals born in Norway at gestational age (GA) <28 weeks or birth weight (BW) <1,000 gram during 1999–2000 (15). The inclusion process, data collection, and outcome at discharge from the neonatal intensive care unit (NICU) have been described in previous reports (16). PDA surgery was performed at four different hospitals. The indication for surgery was determined at the discretion of the neonatologists responsible for neonatal care and was based on clinical signs and echocardiographic evaluation.

**Figure 1:**
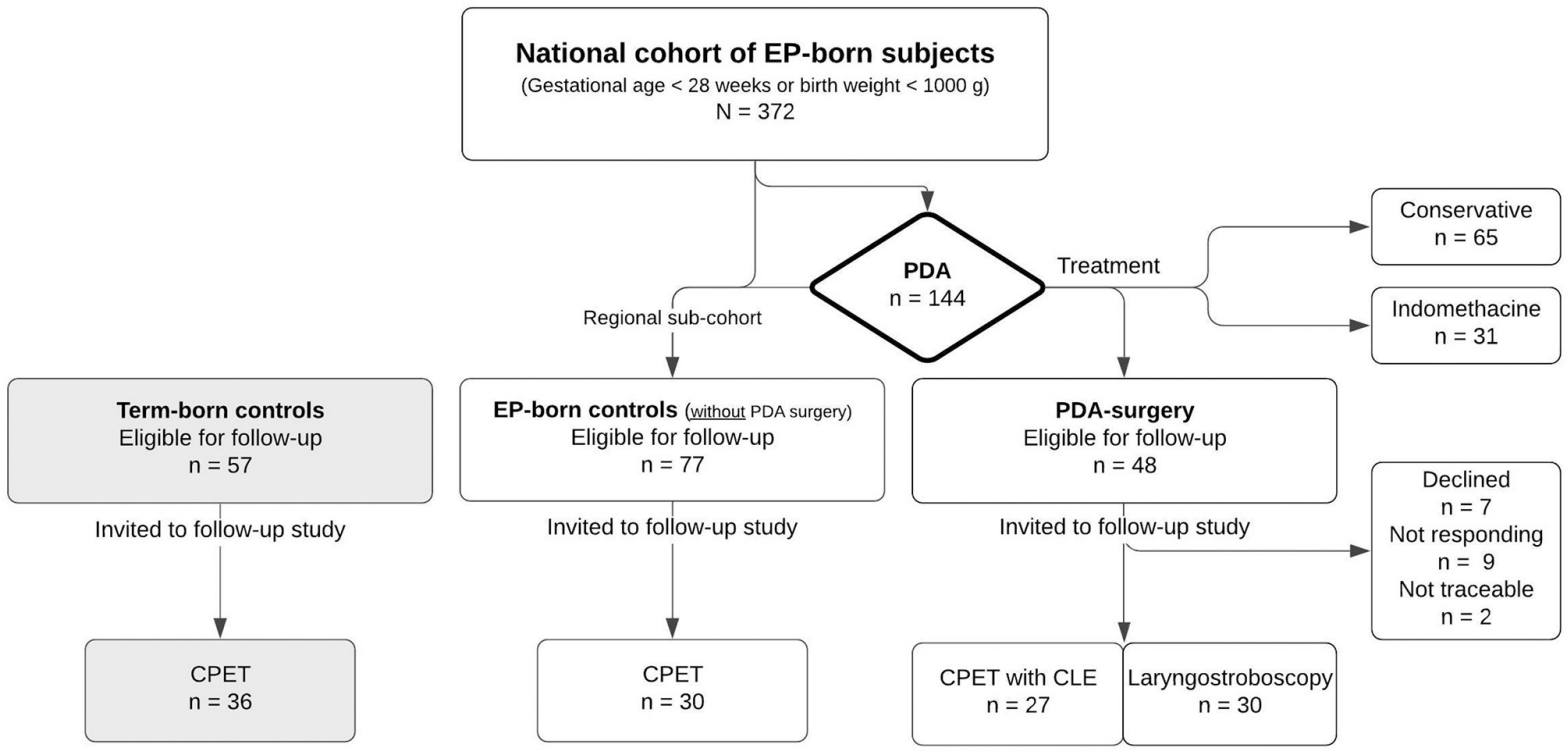
Flow chart of the follow-up study of adults born extremely preterm with a neonatal history of patent ductus arteriosus surgery. CLE, continuous laryngoscopy exercise; CPET, cardiopulmonary exercise test; EP, extremely preterm (gestational age <28 weeks or birth weight <1,000 g); PDA: patent ductus arteriosus.

The present study was conducted during 2018–2020, enrolling three groups (Figure 1):

(1) PDA-surgery: All individuals who had undergone neonatal PDA surgery and were enrolled in the nationwide cohort described above. This PDA-surgery group has two subgroups: those with and those without LVCP.
(2) EP-born controls: A regional sub-sample (Western Norway) of the same nationwide cohort from which the PDA-surgery group was recruited; however, with no history of neonatal PDA surgery.
(3) Term-born controls: At 11 years of age, term born children were recruited as controls for the regional subsample of the EP-born children. The term born children were identified from birth protocols at the maternity ward and were invited as the next-born child of the same gender as the EP born child, with GA >37 weeks and BW >3,000 grams, corresponding to the Norwegian 10th-centile for BW.

### Pulmonary Function

Vyntus^®^ PNEUMO spirometer (Vyaire Medical GmbH, Leibnizstrasse, Hoechberg, Germany) was used to perform spirometry according to guidelines (17). Forced vital capacity (FVC), forced expiratory volume in 1 second (FEV_1_) and FEV_1_/FVC were recorded. Raw data were transformed to z-scores using the reference equations of the Global Lung Function Initiative (18).

### Cardiopulmonary Exercise Test

Peak exercise capacity was determined using a computerized incremental treadmill (Woodway PPS 55 Med, Weil am Rhein, Germany) exercise test according to a modified Bruce protocol (19) using a Vyntus CPX unit powered by SentrySuite software (Vyaire Medical GmbH, Hoechberg, Germany). Speed and elevation were increased every 90 s from an initial slow-walking phase. The test was stopped when the subject indicated severe exhaustion, preferably supported by a respiratory exchange ratio (RER) exceeding 1.05 or heart rate exceeding 95% of predicted maximal heart rate (20).

Variables of gas exchange and airflow were measured breath by breath and averaged over 10 s. The highest values for oxygen uptake determined during the last 60 s were recorded as peak values (VO_2_peak). VO_2_peak was reported as ml/kg/min and as the percentage of predicted using reference equations from a large sample of Norwegian subjects of relevant age (21). Exercise performance was described by the completed distance (meters) on the treadmill. The percentage inspiratory time to total time in a respiratory cycle (T_i_/T_tot_%) was used to describe the breathing pattern. Breathing reserve was the difference between maximal voluntary ventilation (FEV_1_ x 35) and peak minute ventilation reported as the percentage of maximal voluntary ventilation.

### Continuous Laryngoscopy Exercise (CLE) Test

CPET in the PDA-surgery group was performed with concomitant continuous transnasal flexible video-laryngoscopy (ENF TYPE V2, video processor CV-170, OLYMPUS, Tokyo, Japan) as described previously (22). LVCP was identified and later verified by laryngeal stroboscopy. The video recordings of the laryngeal inlet during treadmill running were assessed and rated for laryngeal obstruction according to a modified version of the classification described by Maat et al. (23). Because of laryngeal asymmetry in subjects with LVCP, a modified CLE-score (0–24 points) was developed, assessing the right and left glottic and supraglottic areas separately. The visually assessed medial rotation of the aryepiglottic folds and medialization of the vocal folds were scored ranging from normal (0 points) to maximal (3 points) at moderate (fast walking) and at maximal effort. The left and right sides were scored separately. The total modified CLE-score was the sum of the sub-scores at moderate and maximal exercise.

### Questionnaires

All participants were asked to complete an online questionnaire mapping several health issues. The PDA-surgery group filled in an additional paper-based questionnaire adapted from the Voice Handicap Index with questions regarding voice symptoms (24). The question on physical activity was adapted from the European Community Respiratory Health Survey II questionnaire (25). The questions “*Do you have breathing problems beyond normal during physical exertion*?” *Do you make scraping sounds or other abnormal sounds from the throat during physical exertion? Is your voice hoarser than in others of the same age?* and “*Does your voice affect participation in singing?”* were custom-made for the project.

### Statistical Methods

Data were analyzed using the statistical software SPSS version 26 (IBM SPPS Statistics, NY, USA) and MedCalc version 19.5.3 (MedCalc Software Ltd, Osted, Belgium). Group comparisons were performed using the independent samples *t*-tests (equal variance not assumed) with 95% confidence intervals (95% CI), Mann-Whitney *U*-tests, or Fisher's exact tests, as appropriate. Analysis of covariance was used when the outcome for completed distance and VO_2_peak was adjusted for gender and self-reported physical activity (hours of exercise per week) and to adjust for bronchopulmonary dysplasia (BPD) when comparing lung function variables between the PDA-surgery group and the EP-born control group. To examine whether the difference in VO_2_peak between all EP-born and term-born controls differed by gender, an interaction term for gender and group affiliation was included. Linear regression with the modified CLE-score and gender as predictors was used to investigate whether VO_2_peak was associated with the CLE-score after adjusting for gender. *P*-values ≤ 0.05 was characterized as statistically significant.

### Ethics

The Regional Committee for Medical and Health Research Ethics in Western Norway approved the study. Informed written consents were obtained from all participants, or their parents if subjects were not competent to give consent.

## Results

Thirty of 48 (63%) eligible subjects in the nationwide PDA-surgery cohort consented to participate (Figure 1). One participant was unable to perform spirometry, and two were unable to run on the treadmill because of neurodevelopmental disability. Neonatal and demographic characteristics are given in Tables 1, 2.

**Table 1:** Early characteristics of the extremely preterm born adults enrolled in the national follow-up study on long-term consequences of neonatal patent ductus arteriosus surgery.

	**PDA-surgery** **assessed**		**PDA-surgery** **not assessed**			**EP-born** **controls**		
**Characteristics**	***n*** **= 30**		***n*** **= 18**		***p*** **^a^**	***n*** **= 30**		***p*** **^b^**
Female gender, *n* (%)	14	(47)	4	(22)	0.13	17	(57)	0.61
Birthweight, grams, mean (SD)^1^	792	(178)	781	(169)	0.83	845	(165)	0.24
Age of gestation, weeks, median (range)^2^	26	(23–29)	25	(23–27)	0.94	27	(24–31)	<0.001
Small for gestational age, *n* (%)	4	(13)	3	(17)	1.00	13	(43)	0.02
Prenatal steroids, *n* (%)	20	(67)	13	(72)	0.76	27	(90)	0.06
Surfactant, *n* (%)	27	(90)	18	(100)	0.28	24	(80)	0.47
Postnatal steroids, *n* (%)	20	(67)	14	(78)	0.52	8	(27)	0.004
Invasive ventilation, *n* (%)	29	(97)	17	(94)	1.00	25	(83)	0.20
Invasive ventilation, days, median (range)^2^	13	(1–87)	24	(1–52)	0.65	4	(1–21)	0.003
CPAP treatment, days, median (range)^2^	28.5	(0–92)	18	(4–58)	0.33	26	(0–72)	0.53
Patent ductus arteriosus, *n* (%)	30	(100)	18	(100)	1.00	11	(37)	<0.001
Age patent ductus arteriosus surgery, median (range)^2^	11	(4–34)	10	(2–36)	0.61	–	–	–
Bronchopulmonary dysplasia, *n* (%)	24	(80)	15	(83)	1.00	11	(37)	0.001
Normal neonatal cerebral ultrasound, *n* (%)	18	(60)	5	(28)	0.04	24	(80)	0.16

*CPAP, continuous positive airway pressure; EP, Extremely preterm (gestational age <28 weeks or birthweight <1,000 g); PDA: patent ductus arteriosus. Bronchopulmonary dysplasia defined by oxygen supply and/or ventilatory support at gestational age 36 weeks. Prenatal steroids were recorded if given at least 24 h before delivery. Small for gestational age was defined as under the 10th percentile for gestational age (26). p) Fisher's exact test were used unless*

1 *independent t-test (equal variance not assumed) or*

2 *Mann-Whitney U-test is specified.*

a *Differences between the group of subjects assessed and not assessed among those who had undergone PDA surgery;*

b *Differences between the assessed PDA-surgery group and EP-born controls*.

**Table 2:** Comparison of demographic and anthropometric variables between the groups of extremely preterm born subjects with- or without LVCP, EP-born controls and term-born controls.

			**PDA-surgery**					**EP-born controls**			**Term-born controls**			
	**Total** ***n*** **= 27** **(12 females)**		**LVCP** ***n*** **= 14 (5 females)**		**No LVCP** ***n*** **= 13** **(7 females)**			***n*** **=30** **(17 females)**			***n*** **=36** **(13 females)**			
**Variables**	**Mean**	**SD**	**Mean**	**SD**	**Mean**	**SD**	***p*** **^a^**	**Mean**	**SD**	***p*** **^b^**	**Mean**	**SD**	***p*** **^c^**	***p*** **^d^**
Age, years	19.4	0.7	19.3	0.7	19.5	0.8	0.40	20.4	1.0	<0.001	20.2	1.0	0.001	0.38
Height, *cm*	169.4	9.1	171.1	10.1	167.5	7.9	0.31	167.2	8.9	0.37	177.5	9.7	0.001	<0.001
Females	163.4	6.1	164.0	5.3	163.1	7.0	0.80	162.1	5.7	0.55	168.1	8.9	0.14	0.05
Males	174.1	8.4	175.1	10.1	172.8	5.6	0.58	173.9	7.9	0.95	182.8	5.0	<0.001	0.002
Weight*, kg*	63.8	12.7	65.5	12.4	61.9	13.2	0.47	65.4	16.5	0.68	73.7	14.7	0.006	0.04
Females	61.3	11.6	62.1	15.2	60.7	9.6	0.85	58.2	12.9	0.52	66.2	16.1	0.39	0.16
Males	65.8	13.5	67.4	11.0	63.4	17.4	0.63	74.8	16.3	0.13	77.9	12.3	0.009	0.55
BMI, *kg/m^2^*	22.1	3.5	22.3	3.5	21.9	3.7	0.80	23.3	5.3	0.33	23.3	3.6	0.22	0.97
Females	22.8	3.4	22.9	4.7	22.7	2.5	0.92	22.2	5.4	0.75	23.2	4.0	0.78	0.58
Males	21.6	3.7	22.0	2.9	21.0	4.8	0.69	24.7	4.9	0.08	23.3	3.4	0.17	0.38

*BMI, body mass index; EP, Extremely preterm (gestational age <28 weeks or birthweight <1,000 g); LVCP, left vocal cord paralysis; PDA, patent ductus arteriosus. p) Independent sample t-test (equal variance not assumed);*

a *LVCP vs. no LVCP;*

b *PDA-surgery group vs. EP-born controls;*

c *PDA-surgery group vs. term-born controls;*

d *EP-born controls vs. term-born controls*.

### Left Vocal Cord Paralysis

In the PDA-surgery group, sixteen (53%) subjects were diagnosed with LVCP. Two subjects (7%) had laryngeal stenosis in addition to LVCP, and one subject (3%) presented right-sided arytenoid prolapse with overlying left-sided arytenoid fold making vocal cord assessment during phonation difficult, and LVCP could therefore not be determined (these three subjects are referred to as *other pathology* and they were excluded from further analysis). Thirteen subjects (43%) had a normal laryngeal exam (no LVCP or major anatomic pathology). One subject with LVCP and all three subjects with *other pathology* were aware of their laryngeal pathology before entering this study, the remaining 12 were not. Within the PDA-surgery group, those with LVCP had more often received postnatal steroids compared to those with a normal larynx, whereas other neonatal characteristics were similar (Supplementary File).

Only 14% of those with LVCP compared to 69% of those without LVCP reported no voice-related symptoms (*p* = 0.006) (Table 3). Around 50% reported abnormal sounds from the throat and breathing problems during physical exertion, with no differences between the groups with and without LVCP. All three subjects with *other pathology* reported voice symptoms and breathing problems during physical exertion.

**Table 3:** Self-reported respiratory- and voice symptoms between groups of adults born EP with- or without LVCP and EP-born controls.

	**PDA-surgery**				**EP-born controls**	
**Symptoms**	**LVCP** ***N* = 14**	**No LVCP** ***N* = 13**	**OP** ***N* = 3**	***p*** **^a^**	***N* = 23**	***p*** **^b^**
Hoarse voice, *n* (%)	8 (57)	1 (8)	3 (100)	0.01	3 (13)	0.09
Voice affects participation in singing, *n* (%)	8 (57)	1 (8)	3 (100)	0.01	–	
Voice that cracks when shouting, *n* (%)	7 (50)	2 (15)	3 (100)	0.10	–	
Weak or unclear voice which limits the possibility for being heard in a noisy environment, *n* (%)	8 (57)	4 (31)	3 (100)	0.25	–	
Voice affects participation in school-work or social activities, *n* (%)	4 (29)	3 (23)	3 (100)	1.00	–	
None of the symptoms above, *n* (%)	2 (14)	9 (69)	0 (0)	0.006	–	
Asthma medications last 12 months, *n* (%)	3 (21)	1 (8)	1 (33)	0.60	4 (17)	1.00
Breathing problems beyond normal during normal physical exertion, *n* (%)	9 (64)	6 (46)	3 (100)	0.45	7 (30)	0.09
“Scraping” sound or abnormal sounds during physical exertion, *n* (%)	6 (42)	2 (15)	2 (67)	0.21	2 (9)	0.09

*EP, extremely preterm (gestational age <28 weeks and/or birthweight <1,000 g); LVCP, left vocal cord paralysis; OP, other pathology; PDA, patent ductus arteriosus. p) Fisher's exact test*

a *LVCP vs. no LVCP;*

b *PDA-surgery group (LVCP + no LVCP) vs. EP-born controls*.

### Lung Function

The three participants with *other pathology* were excluded from the analyses of lung function and exercise capacity. Within the PDA-surgery group, we did not find statistically significant differences in spirometry values between subjects with or without LVCP. However, clinically relevant differences could not be excluded given the wide the confidence intervals (Table 4).

**Table 4:** Comparison of spirometry results between groups of adults born EP with- or without LVCP, EP-born controls and term-born controls.

	**PDA-surgery**							**EP-born controls**			**Term-born controls**			
	**Total** ***N*** **= 6** **(12 females)**		**LVCP** ***n*** **= 13** **(5 females)**		**No LVCP** ***n*** **= 13** **(7 females)**			***n*** **= 30** **(17 females)**			***n*** **= 36** **(13 females)**			
**Variables**	**Mean**	**95%CI**	**Mean**	**95%CI**	**Mean**	**95%CI**	***p*** **^a^**	**Mean**	**95%CI**	***p*** **^b^**	**Mean**	**95%CI**	***p*** **^c^**	***p*** **^d^**
FVC, *L*	4.11	3.68, 4.55	4.32	3.67, 4.97	3.90	3.26, 4.55	0.34	4.32	3.95, 4.68	0.46	5.17	4.80, 5.53	<0.001	0.001
FVC, *z-score*	−0.92	−1.44 to −0.40	−0.80	−1.35 to −0.25	−1.05	−2.02, 0.07	0.64	−0.16	−0.49 to 0.18	0.02	−0.10	−0.32 to 0.12	0.005	0.77
FEV_1_, *L*	3.10	2.76, 3.44	3.25	2.69, 3.80	2.96	2.49, 3.42	0.39	3.49	3.21, 3.76	0.08	4.31	4.03, 4.59	<0.001	<0.001
FEV_1_, z–score	−1.76	−2.31 to −1.21	−1.79	−2.52 to −1.06	−1.73	−2.68 to −0.79	0.92	−0.68	−1.07 to 0.29	0.002	−0.28	−0.51 to −0.04	<0.001	0.08
FEV_1_ /FVC ratio	0.76	0.72, 0.80	0.75	0.69, 0.82	0.77	0.71, 0.83	0.74	0.81	0.79, 0.84	0.03	0.84	0.82, 0.86	<0.001	0.17
FEV_1_ /FVC, z-score	−1.50	−2.01 to −0.98	−1.52	−2.36 to −0.68	−1.47	−2.19 to −0.74	0.92	−0.81	−1.20 to −0.42	0.03	−0.36	−0.61 to −0.11	<0.001	0.06

*EP, Extremely preterm (gestational age <28 weeks or birthweight <1,000 g); FEV_1_: forced expiratory in 1 s; FVC, forced vital capacity; LVCP: left vocal cord paralysis; PDA: patent ductus arteriosus; 95%CI: 95% confidence interval. p) Independent sample t-test (equal variance not assumed);*

a *LVCP vs. no LVCP;*

b *PDA-surgery group vs. EP-born controls;*

c *PDA-surgery group vs. term-born controls;*

d *EP-born controls vs. term-born controls*.

**Figure 2:**
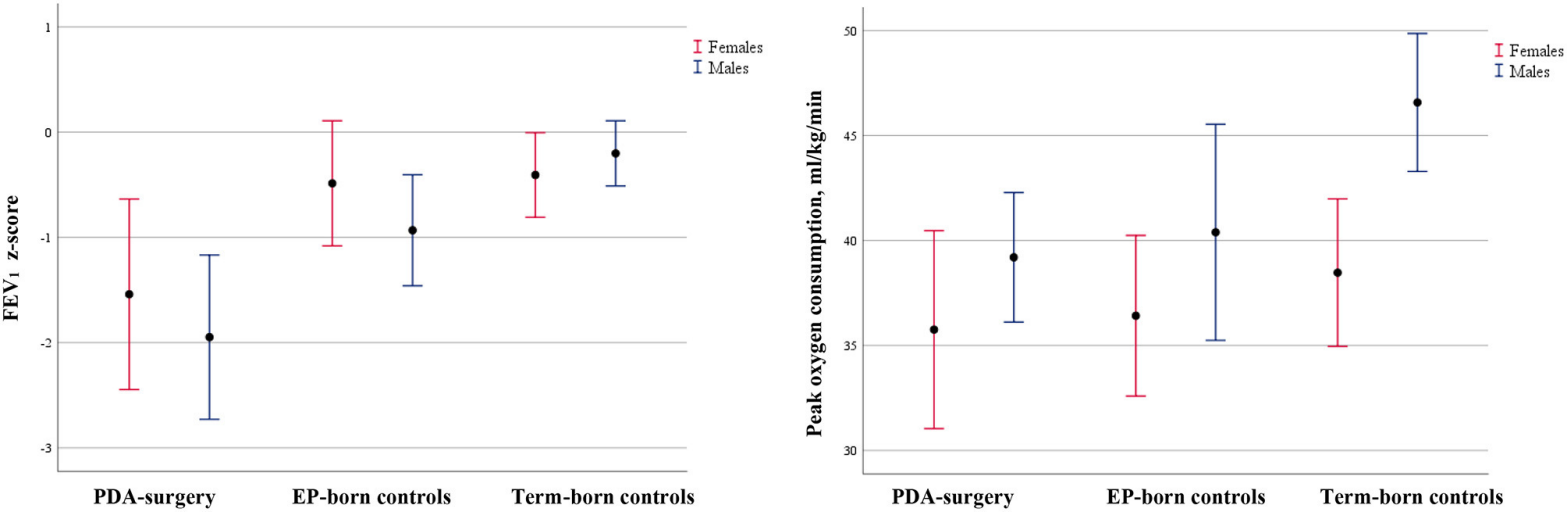
Comparison of lung function and oxygen consumption between EP adults who underwent neonatal patent ductus arteriosus surgery, EP-born controls, and term-born controls. Error bars of mean with 95% CI for FEV_1_ z-score and peak oxygen consumption (ml/kg/min) for the PDA-surgery group, EP-born controls, and term-born controls stratified by gender. *Abbreviations*: EP: extremely preterm (gestational age <28 weeks and/or birth weight <1,000 g); PDA: patent ductus arteriousus.

The PDA surgery group had reduced z-FVC, z-FEV_1_, and z-FEV_1_/FVC, compared to the EP-born controls and the term-born controls (Table 4; Figure 2; Supplementary File). Neonatal BPD was present in 80% of the PDA-surgery group and in 37% of the EP-born controls, and BPD was associated with reduced z-FVC and z-FEV_1_. Adjusting for BPD, z-FEV_1_ was still significantly lower in the PDA-surgery group compared to the EP-born group with a mean (95%CI) difference of 0.89 (1.17, 1.61), *p* = 0.02.

### Exercise Capacity

All participants ran to perceived maximal exhaustion and all achieved RER above 1.05 or heart rate above 95% predicted. Within the PDA-surgery group, we did not find statistically significant differences in completed distance, VO_2_peak (ml/kg/min as well as the percentage of predicted), or self-reported physical activity for subjects with and without LVCP (Table 5; Supplementary File). However, clinically relevant differences could not be excluded given the wide confidence intervals. T_i_/T_tot_% was higher in the participants with LVCP compared to those without LVCP, and also higher than in the EP-born controls and the term-born controls. Mean (95% CI) difference between those with LVCP vs. all the other groups combined was 2.8% (1.6, 4.1) *p* < 0.001.

**Table 5:** Comparison of cardiopulmonary exercise measures in the group of adults born EP with- or without LVCP, EP-born controls and term-born controls.

	**PDA-surgery**							**EP-born controls**			**Term-born controls**			
	**Total** ***N*** **= 25** **(12 females)**		**LVCP** ***n*** **= 13** **(5 females)**		**No LVCP** ***n*** **= 12** **(7 females)**			***n*** **= 30** **(17 females)**			***n*** **= 36** **(13 females)**			
**CPET variables**	**Mean**	**95%CI**	**Mean**	**95%CI**	**Mean**	**95%CI**	***p*** **^a^**	**Mean**	**95%CI**	***p*** **^b^**	**Mean**	**95%CI**	***p*** **^c^**	***p*** **^d^**
Peak heart rate, *beat/min*	191	185, 197	191	181, 201	191	183, 199	0.98	193	190, 196	0.54	195	191, 198	0.27	0.43
RER at peak exercise, units	1.24	1.21, 1.28	1.22	1.16, 1.27	1.27	1.22, 1.31	0.16	1.27	1.23, 1.30	0.24	1.26	1.24, 1.28	0.36	0.65
Ti/Ttot, %	51.0	49.9, 52.1	52.4	51.2, 53.6	49.4	47.8, 51.0	0.004	49.7	48.7, 50.7	0.09	49.5	48.6, 50.3	0.03	0.75
Breathing reserve, %	17	11, 23	20	13, 28	13	3, 23	0.19	16	11, 22	0.93	11	6, 16	0.12	0.11
Peak respiratory rate, *breaths/min*	46	42, 50	43	38, 48	49	42, 55	0.13	48	44, 51	0.47	54	50, 58	0.003	0.01
Females	47	41, 53	44	35, 53	50	40, 60	0.27	46	42, 51	0.76	51	45, 57	0.36	0.19
Males	44	39, 50	43	35, 50	47	34, 61	0.42	49	44, 54	0.18	56	50, 61	0.004	0.06
Peak minute ventilation, *L/min*	89	74, 99	90	74, 105	89	75, 103	0.94	101	92, 110	0.07	134	123, 144	<0.001	<0.001
Females	77	67, 86	72	51, 93	80	66, 94	0.43	90	80, 99	0.07	102	93, 110	<0.001	0.03
Males	101	86, 115	101	80, 121	101	68, 135	0.95	118	106, 129	0.06	152	141, 162	<0.001	<0.001
Distance, *meter*	892	805, 978	935	783, 1,086	835	779, 890	0.19	858	763, 953	0.59	1,117	1,017, 1,216	0.001	<0.001
Females	856	627, 1,084	932	305, 1,558	780	682, 878	0.50	777	667, 887	0.49	917	810, 1,024	0.59	0.06
Males	914	840, 988	936	809, 1,063	879	820, 937	0.35	964	801, 1,127	0.55	1,230	1,104, 1,355	<0.001	0.01
Peak VO_2_, *ml/kg/min*	37.5	34.9, 40.2	38.5	33.6, 43.4	36.5	33.9, 39.0	0.43	38.1	35.1, 41.1	0.76	43.6	41.0, 46.5	0.002	0.007
Females	35.8	31.0, 40.5	38.1	23.9, 52.3	34.1	31.8, 36.4	0.48	36.4	32.6, 40.2	0.82	38.5	35.0, 42.0	0.32	0.40
Males	39.2	36.1, 42.3	38.8	33.7, 43.9	39.8	35.6, 44.0	0.70	40.4	35.2, 45.5	0.67	46.6	43.3, 49.9	0.001	0.04
Peak VO_2_, *% of predicted*	79.6	73.5, 85.8	80.3	68.1, 92.4	79.0	74.9, 79.3	0.83	83.1	76.5, 89.6	0.44	90.2	85.5, 94.9	0.007	0.08
Females	85.0	73.6, 96.4	90.6	56.3, 124.9	81.0	75.5, 86.6	0.49	87.4	78.1, 96.6	0.73	92.2	83.9, 100.4	0.28	0.41
Males	74.7	68.8, 80.5	73.8	64.1, 83.5	76.1	68.3, 83.9	0.65	77.4	67.8, 87.1	0.60	89.1	82.9, 95.3	0.001	0.04

*CPET, cardiopulmonary exercise test; EP, Extremely preterm (gestational age <28 weeks and/or birthweight <1,000 g); LVCP, left vocal cord paralysis; PDA, patent ductus arteriosus; RER, respiratory exchange ratio; Ti/Ttot, Inspiratory time/Total inspiratory and expiratory time ratio; VO_2_, oxygen consumption; 95% CI, 95% confidence interval. Breathing reserve is the difference between maximal voluntary ventilation (FEV_1_ x 35) and peak minute ventilation reported as the percentage of maximal voluntary ventilation. (p) Independent sample t-test (equal variance not assumed);*

a *LVCP vs. no LVCP;*

b *PDA-surgery group vs. EP-born controls;*

c *PDA-surgery group vs. term-born controls;*

d *EP-born controls vs. term-born controls*.

**Figure 3:**
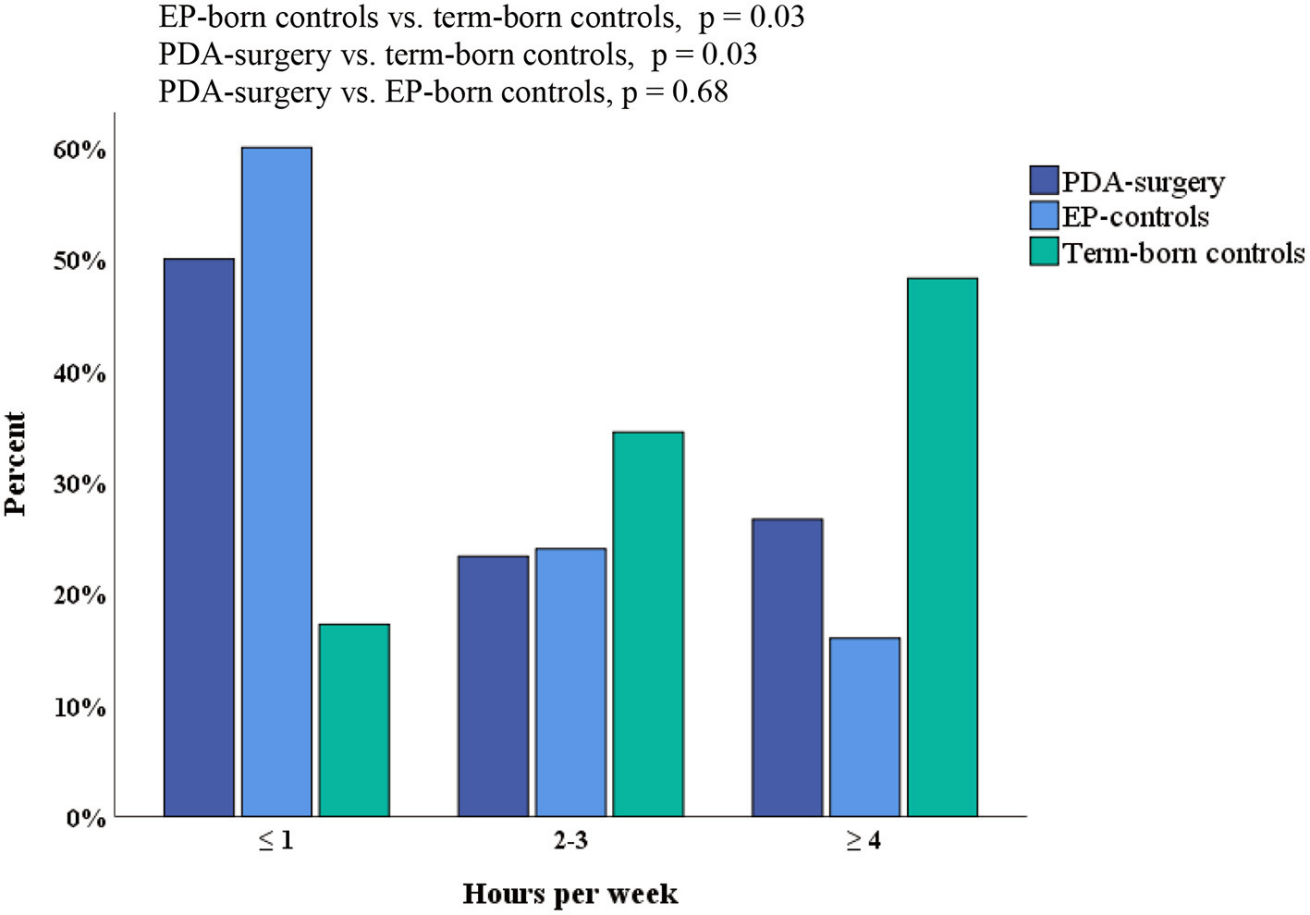
Self-reported physical activity among the EP-born participants who underwent neonatal PDA surgery, EP-born controls and term-born controls. Answer to the self-reported question “*How many hours per week do you attend sports, exercise, or exert yourself so much that you get out of breath and/or sweat?”* Response rate: PDA-surgery: *n* = 30/30, EP-born controls: *n* = 25/30, Term-born controls: *n* = 29/36 p) Chi-square test. EP, extremely preterm (gestational age <28 weeks and/or birth weight <1,000 g); PDA, patent ductus arteriousus.

The PDA-surgery group had similar exercise capacity and self-reported physical activity as the EP-born control group. All EP-born participants combined (PDA-surgery and EP-born controls), ran a shorter distance, had lower VO_2_peak (ml/kg/min), and reported less physical activity compared to term-born controls (Table 5; Figures 2, 3; Supplementary File). Adjusted for gender, mean (95% CI) difference in completed distance and VO_2_peak between all the EP-born participants combined vs. the term-born controls was 218 (114, 322) meters, *p* < 0.001, and 4.9 (1.8, 8.0) ml/kg/min, *p* = 0.002, respectively. There was no significant interaction effect between gender and group affiliation (all EP-born and term-born) on VO_2_peak (*p* = 0.16). After additional controlling for physical activity, the completed distance on the treadmill was still shorter for EP-born participants compared to the term-born control group [mean (95% CI) difference 150 (39, 260) meters, *p* = 0.009]. Moreover, VO_2_peak difference was slightly reduced [3.2 (−0.2, 6.7) ml/kg/min] and no longer statistically significant (*p* = 0.07).

### Continuous Laryngoscopy Exercise Findings (PDA-Surgery Participants Only)

In the PDA-surgery group, 27/30 participants performed a CLE test. Among these, the modified CLE-score at moderate and maximal effort could not be determined in three subjects, and the total score was derived from the sub-scores at rest and maximal effort, or at rest and moderate effort.

**Figure 4:**
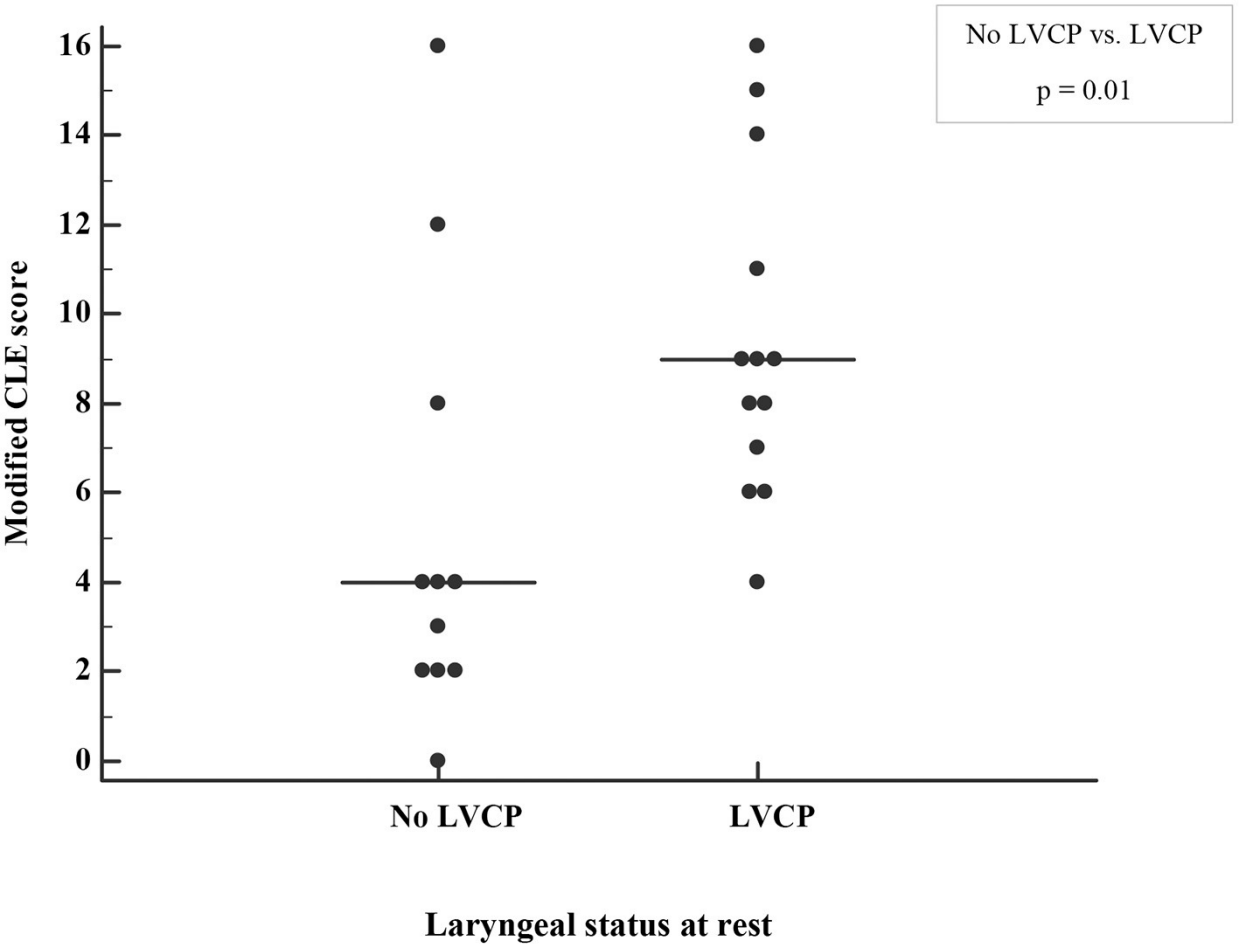
Laryngeal obstruction during exercise graded by a modified continuous laryngoscopy exercise (CLE) score in the adults born EP with or without left vocal cord paralysis (LVCP). The median CLE-scores are indicated by horizontal lines. p) Mann-Whitney *U*-test.

**Figure 5:**
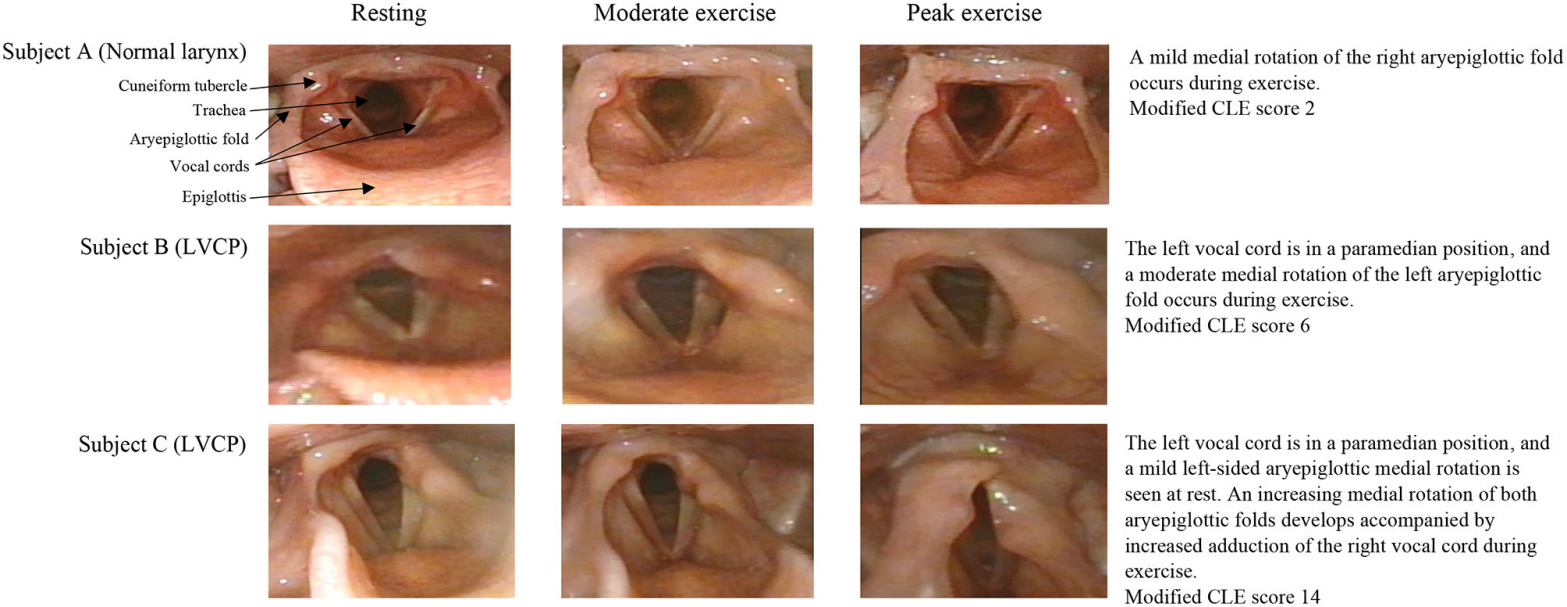
Images of the larynx during exercise in EP-born individuals who had undergone neonatal PDA surgery.

In the group with LVCP, all but one had a modified CLE-score >4, indicating laryngeal obstruction during exercise. In those with no LVCP, only three subjects had a modified CLE-score >4 (Figure 4), which suggests they had the specific diagnosis of EILO as all had normal larynx at rest (27). Figure 5 demonstrates the laryngeal inlet in three participants, one with a normal larynx and two with LVCP.

The group of subjects reporting breathing difficulties during exercise and those reporting abnormal sounds from the throat during exercise did not have a higher modified CLE-score than those without these symptoms (Supplementary File). Furthermore, the modified CLE-score was not associated with VO_2_peak after adjusting for gender (*p* = 0.40).

## Discussion

This is the first study to report the prevalence of LVCP in a national cohort of young adults with a history of EP birth and surgical closure of PDA during their neonatal period. Within the PDA-surgery group, more than half of the participating subjects were diagnosed with LVCP, which was associated with characteristic voice symptoms, prolonged inspiratory to total respiratory time, and laryngeal obstruction during exercise. We did not find an association between LVCP and lung function or exercise capacity, however, the power to detect such associations was low due to the limited sample size. Overall, the PDA-surgery group had impaired lung function compared to EP-born as well as term-born controls, whereas exercise capacity was similarly reduced for both PDA-surgery and EP-born controls compared to term-born controls.

Studies examining LVCP after PDA surgery in EP-born neonates have mainly been performed in the immediate postoperative period or during infancy (28). Our group has previously identified LVCP in 7 of 11 EP-born adults participating in a small local cohort study (12). In this national study, the prevalence of LVCP was 53%, compared to the 32% reported in a meta-analysis of studies examining all infants after PDA surgery (28). Performing laryngeal examinations on neonates is challenging, and pathology may be overlooked (29). This may explain the high prevalence of LVCP in studies assessing adults who are easier to examine. We do not have complete information on whether clips or ligature were used for PDA closure; hence, we could not assess a possible influence from the mode of surgery.

Dysphonia is a known long-term complication of preterm birth and is associated with extreme prematurity, emergency intubations, and multiple intubations, as well as PDA surgery (13, 30, 31). Unilateral vocal cord paralysis is associated with social and physical limitations and reduced health-related quality of life (32). There were more reports of voice symptoms in the subjects with LVCP; however, about one-third of subjects without LVCP also reported voice symptoms. Several of the subjects with LVCP in our study reported that their voice affected their participation in singing, social activities, and schoolwork. Surgical treatment and voice therapy may improve voice quality, and we encourage that a laryngeal examination is performed after neonatal PDA surgery (33).

Local traumas related to intubation and prolonged time on invasive mechanical ventilation are known risk factors for laryngeal injury (34). We found that 3/30 (10%) of the participants who underwent laryngoscopy had major laryngeal pathology other than LVCP. All three reported voice symptoms and breathing problems during exercise and all were aware of their malfunctioning larynx prior to study enrollment, contrasting the participants with LVCP, where only one had been aware of their pathology in advance. This certainly underlines the importance of suspecting laryngeal pathology in EP-born individuals with voice or respiratory complaints and to include an upper airway assessment to achieve a comprehensive understanding of their symptoms.

### BPD and PDA

In the PDA-surgery group in this present study, more had BPD and the lung function was poorer in adulthood, compared to the EP-born control group. There is convincing evidence that preterm-born survivors with or without BPD have an increased risk for poor adult lung function (35). The association between PDA surgery and BPD has been reported earlier (8, 36), and may be explained by more severe neonatal respiratory illness, as PDA surgery tends to be performed as “rescue therapy” in infants with already advanced lung disease and/or failed pharmacological treatment of PDA (5, 37). However, population-based observational studies have suggested that early surgical ligation is an independent risk factor for BPD (38, 39). A re-examination of the only randomized controlled trial investigating the effects of prophylactic PDA ligation vs. delayed ligation revealed a significant increase in BPD incidence in those who were ligated prophylactically (40). Animal studies support a link between PDA ligation and the development of chronic lung disease by increased expression of genes involved in pulmonary inflammation and decreased alveolar fluid clearance (41). However, these issues are incompletely understood.

### Exercise Capacity, Physical Activity, and Laryngeal Obstruction During Exercise

In individuals with LVCP, the para-median position of a paralyzed left vocal cord would be expected to interfere with the normal exercise-induced dilation of the glottis, and thus potentially compromise airflow capacity and exercise capacity. We found that subjects with LVCP had prolonged inspiration and a tendency for a lower peak respiratory rate at peak exercise. By laryngoscopy, we observed severe laryngeal obstruction during exercise in several individuals affected with LVCP (Figure 5). However, LVCP and the modified CLE-score were not associated with VO_2_peak. This finding is in line with our previous study, where no association between VO_2_peak and LVCP was found in EP-born adults (12). The results from these two studies suggest that it is possible to obtain average exercise capacity despite a relatively severe laryngeal obstruction.

A number of long-term sequelae of EP birth may affect subsequent exercise capacity, such as cardiopulmonary and neuromuscular impairment, reduced skeletal muscle mass, and behavioral issues such as less participation in physical activity (2). A review of 22 studies on exercise capacity concluded that children and adults born preterm have 13% lower VO_2_peak (ml/min/kg) than term-born, in line with the ~11% (-4.9 ml/kg/min) lower VO_2_peak observed for all our EP-born participants combined (14). Similar to previous reports, we found that a lower amount of physical activity may be an explanatory factor for the relatively modest deficit in VO_2_peak (42). It is still not determined if an increased level of physical activity will lead to improved exercise capacity in EP-born adults. Morales Mestre et al. conducted a randomized intervention study on EP-born children diagnosed with BPD and found that a structured exercise program improved exercise capacity (43). We encourage more research to be invested in this area to expand the knowledge on participation in physical activity and trainability in the EP-born population.

### Strengths and Limitations

The strengths of this study were a population-based design with several centers responsible for the PDA surgery, and a high rate of participation. It was a limitation that only the PDA-surgery group was examined with laryngoscopy. Undiscovered LVCP or other laryngeal pathology might have been present in the EP-born control group, due to e.g., pressure from a large PDA or a large pulmonary trunk (44). Furthermore, laryngoscopy was not performed in the neonatal period and preoperative pathology or spontaneous postoperative improvement of LVCP could not be assessed. Cardiopulmonary exercise data for the PDA-surgery group were obtained from CLE-tests, which we have shown can be used interchangeably with data obtained from a regular CPET (22). Information on physical activity was self-reported and not determined by a more objective method like accelerometry or diary. Furthermore, the question on physical activity did not include aspects of mode and intensity, factors that may have affected the correlation between VO_2_peak and physical activity.

The number of eligible subjects was determined by the number of EP-born infants who underwent PDA surgery in Norway during 1999–2000. The sample size was relatively small with large variation within the groups, resulting in a reduced power to detect differences in the subgroup analyses. About one-third of the eligible EP-born adults who had undergone PDA surgery were lost to follow-up (Figure 1). Recruiting young adults with a busy schedule is challenging and individuals with voice or breathing symptoms might have been more motivated to participate than individuals without such symptoms. The estimated prevalence of LVCP in this cohort lies within the range of 16/48 (33%) to 34/48 (71%) if no one or all non-participating subjects were diagnosed with LVCP. Furthermore, the study protocol requested treadmill running which might have motivated those able to and familiar with running to participate. More subjects in the participating group had a normal neonatal cerebral ultrasound compared to the non-participating group, implying a selection of subjects with less neurological sequela (Table 1).

Management of PDA in EP-born individuals is still under debate (45). Reports suggesting associations with negative post-operative outcomes have contributed to a decline in the rate of PDA surgery in the last decade (46). However, selection by indication represents a challenge and may not have been fully accounted for when reporting on outcomes (5, 47). Choice of surgical procedure may also affect outcomes. Surgical ligation has been associated with higher rates of LVCP than surgical clipping (48). Unfortunately, we did not have complete information on surgical methods in our data set. New catheter-based procedures add options for PDA closure also for infants <1,000 g (49). Irrespective of future guidelines for PDA management, a population of EP-born subjects with a history of neonatal PDA surgery already exists. Therefore, clinicians caring for EP-born children and adults should be aware of symptoms and long-term outcomes associated with PDA-surgery and LVCP to ensure proper follow-up.

## Conclusions

In this nationwide study, LVCP was present in 53% of EP-born young adults who had undergone neonatal PDA surgery. Within the PDA-surgery group, LVCP was associated with self-reported voice symptoms and laryngeal obstruction during exercise. We did not find an association between LVCP and lung function and exercise capacity, however; the power to detect such associations was low. Overall, the PDA-surgery group had impaired lung function compared to EP-born and term-born controls, whereas exercise capacity was similarly reduced for both PDA-surgery and EP-born controls compared to term-born controls.

Clinicians caring for EP-born children and adults should be aware of possible laryngeal sequelae after PDA surgery. Furthermore, EP-born subjects with a history of PDA surgery represent a population that needs follow-up to monitor lung function. Despite a high-risk start to life, EP-born individuals who underwent PDA surgery seem to achieve an exercise capacity only modestly decreased compared to term born individuals.

## Data Availability Statement

The datasets presented in this article are not readily available because in accordance with the approvals granted for this study by the Regional Committee on Medical Research Ethics and the Norwegian Data Inspectorate, the data files are stored securely and in accordance with the Norwegian Law of Privacy Protection. The data file cannot be made publicly available as this might compromise the respondents' privacy. Some of the participating centers are small and the number of extremely preterm births is limited with a risk of identifying anonymous participants. A subset of the data file with anonymized data can be made available to interested researchers upon reasonable request, providing Norwegian privacy legislation and GDPR are respected, and that permission is granted from The Norwegian Data Inspectorate and the data protection officer at Haukeland University Hospital. Requests to access the datasets should be directed to Maria Vollsæter, maria.vollseter@helse-bergen.no.

## Ethics Statement

The studies involving human participants were reviewed and approved by the Regional Committees for Medical and Health Research Ethics West, The University of Bergen, 5021 Bergen, Norway. Informed written consents were obtained from all participants, or their parents if subjects were not competent to give consent.

## Author Contributions

ME and MSE have coordinated and collected data, organized data, carried out the analyses, drafted the initial manuscript, and revised the manuscript. MV has designed the data collection instruments, collected and organized data, and has reviewed and revised the manuscript. LS, OG, and IE have collected and organized data and critically reviewed the manuscript for important intellectual content. KH has given advice on the analysis of data, participated in the interpretation of the data, and critically reviewed the manuscript for important intellectual content. KØ, OR, and TH have provided funding, designed the data collection instruments, coordinated and supervised data collection, and have critically reviewed the manuscript for important intellectual content. HC has conceptualized and designed the study, designed the data collection instruments, drafted the initial manuscript, and has critically reviewed the manuscript for important intellectual content. All authors approved the final manuscript as submitted and agree to be accountable for all aspects of the work.

## Funding

This study was financially supported by the Western Norway Regional Health Authority grant number F-11526.

## Conflict of Interest

The authors declare that the research was conducted in the absence of any commercial or financial relationships that could be construed as a potential conflict of interest.

## Publisher's Note

All claims expressed in this article are solely those of the authors and do not necessarily represent those of their affiliated organizations, or those of the publisher, the editors and the reviewers. Any product that may be evaluated in this article, or claim that may be made by its manufacturer, is not guaranteed or endorsed by the publisher.

## References

[1] Fanaroff AA Hack M Walsh MC. The NICHD neonatal research network: changes in practice and outcomes during the first 15 years. Semin Perinatol. (2003) 27:281–7. 10.1016/S0146-0005(03)00055-7 14510318

[2] Raju TNK Buist AS Blaisdell CJ Moxey-Mims M Saigal S. Adults born preterm: a review of general health and system-specific outcomes. Acta Paediatr. (2017) 106:1409–37. 10.1111/apa.13880 28419544

[3] Ngo S Profit J Gould JB Lee HC. Trends in patent ductus arteriosus diagnosis and management for very low birth weight infants. Pediatrics. (2017) 139. 10.1542/peds.2016-2390 28562302 PMC5369670

[4] Koch J Hensley G Roy L Brown S Ramaciotti C Rosenfeld CR. Prevalence of spontaneous closure of the ductus arteriosus in neonates at a birth weight of 1000 grams or less. Pediatrics. (2006) 117:1113–21. 10.1542/peds.2005-1528 16585305

[5] Weisz D More K PJ M PS S. PDA ligation and health outcomes: a meta-analysis. Pediatrics 2014. (2014) 133:e1024. 10.1542/peds.2013-3431 24639268

[6] Jain A Shah PS. Diagnosis, evaluation, and management of patent ductus arteriosus in preterm neonates. JAMA Pediatr. (2015) 169:863–72. 10.1001/jamapediatrics.2015.0987 26168357

[7] Pereira KD Webb BD Blakely ML Cox CS Jr Lally KP. Sequelae of recurrent laryngeal nerve injury after patent ductus arteriosus ligation. Int J Pediatr Otorhinolaryngol. (2006) 70:1609–12. 10.1016/j.ijporl.2006.05.001 16797086

[8] Benjamin JR Smith PB Cotten CM Jaggers J Goldstein RF Malcolm WF. Long-term morbidities associated with vocal cord paralysis after surgical closure of a patent ductus arteriosus in extremely low birth weight infants. J Perinatol. (2010) 30:408–13. 10.1038/jp.2009.124 19759545 PMC2878380

[9] Daya H Hosni A Bejar-Solar I Evans JN Bailey CM. Pediatric vocal fold paralysis: a long-term retrospective study. Arch Otolaryngol Head Neck Surg. (2000) 126:21–5. 10.1001/archotol.126.1.21 10628706

[10] Smith ME King JD Elsherif A Muntz HR Park AH Kouretas PC. Should all newborns who undergo patent ductus arteriosus ligation be examined for vocal fold mobility? Laryngoscope. (2009) 119:1606–9. 10.1002/lary.20148 19507238

[11] Clement WA El-Hakim H Phillipos EZ Cote JJ. Unilateral vocal cord paralysis following patent ductus arteriosus ligation in extremely low-birth-weight infants. Arch Otolaryngol Head Neck Surg. (2008) 134:28–33. 10.1001/archoto.2007.2 18209132

[12] Roksund OD Clemm H Heimdal JH Aukland SM Sandvik L Markestad T. Left vocal cord paralysis after extreme preterm birth, a new clinical scenario in adults. Pediatrics. (2010) 126:e1569–77. 10.1542/peds.2010-1129 21098147

[13] Hseu A Ayele N Kawai K Woodnorth G Nuss R. Voice abnormalities and laryngeal pathology in preterm children. Ann Otol Rhinol Laryngol. (2018) 127:508–13. 10.1177/0003489418776987 29962214

[14] Edwards MO Kotecha SJ Lowe J Watkins WJ Henderson AJ Kotecha S. Effect of preterm birth on exercise capacity: a systematic review and meta-analysis. Pediatr Pulmonol. (2015) 50:293–301. 10.1002/ppul.23117 29889363

[15] Markestad T Kaaresen PI Rønnestad A Reigstad H Lossius K Medbø S. Early death, morbidity, and need of treatment among extremely premature infants. Pediatrics. (2005) 115:1289–98. 10.1542/peds.2004-1482 15867037

[16] Westby Wold SH Sommerfelt K Reigstad H Ronnestad A Medbo S Farstad T. Neonatal mortality and morbidity in extremely preterm small for gestational age infants: a population based study. Arch Dis Child Fetal Neonatal Ed. (2009) 94:F363–7. 10.1136/adc.2009.157800 19439434

[17] Miller MR Hankinson J Brusasco V Burgos F Casaburi R Coates A. Standardisation of spirometry. Eur Res J. (2005) 26:319–38. 10.1183/09031936.05.00034805 16055882

[18] Quanjer PH Stanojevic S Cole TJ Baur X Hall GL Culver BH. Multi-ethnic reference values for spirometry for the 3-95-yr age range: the global lung function 2012 equations. Eur Respir J. (2012) 40:1324–43. 10.1183/09031936.00080312 22743675 PMC3786581

[19] Cumming GR Everatt D Hastman L. Bruce treadmill test in children: normal values in a clinic population. Am J Cardiol. (1978) 41:69–75. 10.1016/0002-9149(78)90134-0 623008

[20] Nes BM Janszky I Wisloff U Stoylen A Karlsen T. Age-predicted maximal heart rate in healthy subjects: The HUNT fitness study. Scand J Med Sci Sports. (2013) 23:697–704. 10.1111/j.1600-0838.2012.01445.x 22376273

[21] Edvardsen E Hansen BH Holme IM Dyrstad SM Anderssen SA. Reference values for cardiorespiratory response and fitness on the treadmill in a 20- to 85-year-old population. Chest. (2013) 144:241–8. 10.1378/chest.12-1458 23287878

[22] Engan M Hammer IJ Bekken M Halvorsen T Fretheim-Kelly ZL Vollsæter M. Reliability of maximum oxygen uptake in cardiopulmonary exercise testing with continuous laryngoscopy. ERJ Open Res. (2020) 2020:00825–2020. 10.1183/23120541.00825-2020 33614778 PMC7882785

[23] Maat RC Roksund OD Halvorsen T Skadberg BT Olofsson J Ellingsen TA. Audiovisual assessment of exercise-induced laryngeal obstruction: reliability and validity of observations. Eur Arch Otorhinolaryngol. (2009) 266:1929–36. 10.1007/s00405-009-1030-8 19585139

[24] Jacobson B Johnson A Grywalski C. The voice handicap index (VHI): development and validation. Am J Speech Lang Pathol. (1997) 6:66–70. 10.1044/1058-0360.0603.6627497397

[25] European Community Respiratory Health Survey IISC. The European community respiratory health survey II. Eur Respir J. (2002) 20:1071–9. 10.1183/09031936.02.00046802 12449157

[26] Skjaerven R Gjessing HK Bakketeig LS. Birthweight by gestational age in norway. Acta Obstet Gynecol Scand. (2000) 79:440–9. 10.1034/j.1600-0412.2000.079006440.x 10857867

[27] Halvorsen T Walsted ES Bucca C Bush A Cantarella G Friedrich G. Inducible laryngeal obstruction: an official joint European respiratory society and European laryngological society statement. Eur Respir J. (2017) 50. 10.1183/13993003.02221-2016 28889105

[28] Engeseth MS Olsen NR Maeland S Halvorsen T Goode A Roksund OD. Left vocal cord paralysis after patent ductus arteriosus ligation: a systematic review. Paediatr Respir Rev. (2018) 27:74–85. 10.1016/j.prrv.2017.11.001 29336933

[29] Ryan MA Upchurch PA Senekki-Florent P. Neonatal vocal fold paralysis. Neo Rev. (2020) 21:e308–22. 10.1542/neo.21-5-e308 32358144

[30] Reynolds V Meldrum S Simmer K Vijayasekaran S French N. Dysphonia in very preterm children: a review of the evidence. Neonatology. (2014) 106:69–73. 10.1159/000360841 24819149

[31] French N Kelly R Vijayasekaran S Reynolds V Lipscombe J Buckland A. Voice abnormalities at school age in children born extremely preterm. Pediatrics. (2013) 131:e733–9. 10.1542/peds.2012-0817 23420908

[32] Fang TJ Li HY Gliklich RE Chen YH Wang PC Chuang HF. Quality of life measures and predictors for adults with unilateral vocal cord paralysis. Laryngoscope. (2008) 118:1837–41. 10.1097/MLG.0b013e31817e7431 18806475

[33] Granato F Martelli F Comini LV Luparello P Coscarelli S Le Seac O. The surgical treatment of unilateral vocal cord paralysis (UVCP): qualitative review analysis and meta-analysis study. Eur Arch Oto Rhino Laryngol. (2019) 276:2649–59. 10.1007/s00405-019-05587-2 31375895

[34] Fan LL Flynn JW Pathak DR. Risk factors predicting laryngeal injury in intubated neonates. Crit Care Med. (1983) 11:431–3. 10.1097/00003246-198306000-00007 6851600

[35] Vollsæter M Røksund OD Eide GE Markestad T Halvorsen T. Lung function after preterm birth: development from mid-childhood to adulthood. Thorax. (2013) 68:767–76. 10.1136/thoraxjnl-2012-202980 23749815

[36] Chorne N Leonard C Piecuch R Clyman RI. Patent ductus arteriosus and its treatment as risk factors for neonatal and neurodevelopmental morbidity. Pediatrics. (2007) 119:1165–74. 10.1542/peds.2006-3124 17545385

[37] Härkin P Marttila R Pokka T Saarela T Hallman M. Morbidities associated with patent ductus arteriosus in preterm infants. Nationwide cohort study. J Mat Fetal Neon Med. (2018) 31:2576–83. 10.1080/14767058.2017.1347921 28651469

[38] Madan JC Kendrick D Hagadorn JI Frantz ID 3rd National National Institute of Child H Human Development Neonatal Research N. Patent ductus arteriosus therapy: impact on neonatal and 18-month outcome. Pediatrics. (2009) 123:674–81. 10.1542/peds.2007-2781 19171637 PMC2752886

[39] Kabra NS Schmidt B Roberts RS Doyle LW Papile L Fanaroff A. Neurosensory impairment after surgical closure of patent ductus arteriosus in extremely low birth weight infants: results from the trial of indomethacin prophylaxis in preterms. J Pediatr. (2007) 150:229–34:34 e1. 10.1016/j.jpeds.2006.11.039 17307535

[40] Clyman R Cassady G Kirklin JK Collins M Philips JB 3rd. The role of patent ductus arteriosus ligation in bronchopulmonary dysplasia: reexamining a randomized controlled trial. J Pediatr. (2009) 154:873–6. 10.1016/j.jpeds.2009.01.005 19324366 PMC2709418

[41] Waleh N McCurnin DC Yoder BA Shaul PW Clyman RI. Patent ductus arteriosus ligation alters pulmonary gene expression in preterm baboons. Pediatr Res. (2011) 69:212–6. 10.1203/PDR.0b013e3182084f8d 21131894 PMC3065199

[42] Clemm H Roksund O Thorsen E Eide GE Markestad T Halvorsen T. Aerobic capacity and exercise performance in young people born extremely preterm. Pediatrics. (2012) 129:e97–105. 10.1542/peds.2011-0326 22201154

[43] Morales Mestre N Papaleo A Morales Hidalgo V Caty G Reychler G. Physical activity program improves functional exercise capacity and flexibility in extremely preterm children with bronchopulmonary dysplasia aged 4-6 years: a randomized controlled trial. Arch Bronconeumol. (2018) 54:607–13. 10.1016/j.arbres.2018.05.001 30518495

[44] Nakahira M Nakatani H Takeda T. Left vocal cord paralysis associated with long-standing patent ductus arteriosus. AJNR Am J Neuroradiol. (2001) 22:759–61. 11290495 PMC7976032

[45] Evans N. Preterm patent ductus arteriosus: a continuing conundrum for the neonatologist? Semin Fetal Neon Med. (2015) 20:272–7. 10.1016/j.siny.2015.03.004 25818393

[46] Reese J Scott TA Patrick SW. Changing patterns of patent ductus arteriosus surgical ligation in the United States. Semin Perinatol. (2018) 42:253–61. 10.1053/j.semperi.2018.05.008 29954594 PMC6512985

[47] Weisz DE McNamara PJ. Patent ductus arteriosus ligation and adverse outcomes: causality or bias? J Clin Neonatol. (2014) 3:67–75. 10.4103/2249-4847.134670 25024972 PMC4089132

[48] Henry BM Hsieh WC Sanna B Vikse J Taterra D Tomaszewski KA. Incidence, risk factors, and comorbidities of vocal cord paralysis after surgical closure of a patent ductus arteriosus: a meta-analysis. Pediatr Cardiol. (2019) 40:116–25. 10.1007/s00246-018-1967-8 30167748 PMC6348263

[49] Vali P Lakshminrusimha S Pelech A Underwood M Ing F. Patent ductus arteriosus in preterm infants: is early transcatheter closure a paradigm shift? J Perinatol. (2019) 39:1449–61. 10.1038/s41372-019-0506-7 31562396

