# Supplementary material for: Left Vocal Cord Paralysis, Lung Function and Exercise Capacity in Young Adults Born Extremely Preterm With a History of Neonatal Patent Ductus Arteriosus Surgery—A National Cohort Study
Source: Front Pediatr. 2022 Jan 3;9:780045. doi: 10.3389/fped.2021.780045 (PMC8761768; doi:10.3389/fped.2021.780045)
Supplement: Supplementary file 1 [file Data_Sheet_1.PDF]

Supplementary Material

Supplementary table 1 Neonatal characteristics of the enrolled extremely preterm born adults who had undergone patent ductus arteriosus surgery

Characteristics	PDA-surgery						
	LVCP n = 14		No LVCP n = 13		Other pathology n = 3		*p*
Female gender, *n (%)*	5	(36)	7	(54)	2	(67)	0.45
Birthweight, *grams, mean (SD)* 1)	767	(174)	820	(197)	786	(144)	0.47
Age of gestation, *weeks, median (range)*2)	25	(23-27)	26	(23-29)	25	(24-26)	0.32
Small for gestational age, *n (%)*	2	(14)	2	(8)	0	(0)	1.00
Prenatal steroids, *n (%)*	9	(64)	9	(69)	2	(67)	1.00
Surfactant, *n (%)*	13	(93)	12	(92)	2	(67)	1.00
Postnatal steroids, *n (%)*	12	(86)	5	(38)	3	(100)	0.02
Invasive ventilation, *n (%)*	14	(100)	12	(92)	3	(100)	0.48
Invasive ventilation, days, *median (range)* 2)	15	(1-85)	10	(2-87)	17	(15-83)	0.30
CPAP treatment, days, *median (range)* 2)	32.5	(0-92)	27	(2-58)	31	(23-50)	0.58
Age PDA surgery, *median (range)* 2)	7.5	(4-31)	11	(4-35)	23	(11-27)	0.31
Bronchopulmonary dysplasia, *n (%)*	12	(86)	10	(77)	2	(67)	0.65
Normal cerebral ultrasound, *n (%)*	7	(50)	9	(69)	2	(67)	0.44

Abbreviations: CPAP: continuous positive airway pressure; EP: extremely preterm (gestational age < 28 weeks or birthweight < 1000 g); LVCP: left vocal cord paralysis; OP: other pathology; PDA: patent ductus arteriosus.

Bronchopulmonary dysplasia defined by oxygen supply and/or ventilatory support at gestational age 36 weeks. Small for gestational age was defined as under the 10th percentile for gestational age. Prenatal steroids were recorded if given at least 24 hours before delivery.

p) Fisher's exact test were used unless 1) independent t-test (equal variance not assumed) or 2) Mann-Whitney U test is specified.

Supplementary table 2 Differences in lung function and cardiopulmonary exercise measures between the group of young adults born EP with- or without LVCP, EP-born controls and term-born control

	**Within PDA-surgery group: LVCP vs. no LVCP**			**PDA-surgery group vs. EP-born controls**			**PDA-surgery group vs. term-born controls**			**EP-born controls vs. term-born controls**		
Spirometry variables	Mean diff	95%CI	p	Mean diff	95%CI	p	Mean diff	95%CI	p	Mean diff	95%CI	p
FVC, *z-score*	0.24	-0.83, 1.32	0.64	-0.77	-1.37, -0.16	0.02	-0.83	-1.39, -0.27	0.005	-0.06	-0.45, 0.34	0.77
FEV_1, z-score	-0.06	-1.19, 1.08	0.92	-1.08	-1.75, -0.42	0.002	-1.48	-2.08, -0.89	<0.001	-0.40	-0.85, 0.05	0.08
FEV_1 /FVC, z-score	-0.05	-1.10, 1.00	0.92	-0.69	-1.32, -0.05	0.03	-1.14	-1.70, -0.57	<0.001	-0.45	-0.91, 0.01	0.06
CPET variables												
Peak heart rate, *beat/min*	0.2	-12.3, 12.6	0.98	-2.0	-8.8, 4.7	0.54	-3.9	-10.8, 3.1	0.27	-1.8	-6.3, 2.7	0.43
RER at peak exercise, units	-0.05	-0.12, 0.02	0.16	-0.03	-0.08, 0.02	0.24	-0.02	-0.06, 0.02	0.36	0.01	-0.03, 0.05	0.65
Ti/Ttot, %	3.0	1.1, 4.9	0.004	1.3	-0.2, 2.7	0.09	1.5	0.1, 2.8	0.03	0.2	-1.1, 1.5	0.75
Breathing reserve, *%*	7.5	-4.1, 19.2	0.19	0.3	-7.2, 7.9	0.93	5.8	-1.5, 13.1	0.12	5.5	-1.3, 12.2	0.11
Peak minute ventilation, *L/min*	0.7	-19.2, 20.6	0.94	-11.9	-24.7, 0.9	0.07	-44.4	-58.4, -30.5	<0.001	-32.5	-45.9, -19.2	<0.001
Females	-7.9	-29.3, 13.6	0.43	-11.9	-25.0, 1.1	0.07	-24.9	-36.7, -13.1	<0.001	-12.9	-24.6, -1.3	0.03
Males	-0.9	-34.8, 33.0	0.95	-16.7	-34.3, 0.9	0.06	-51.0	-67.7, -34.2	<0.001	-34.3	-48.4, -20.2	<0.001
Distance, *meter*	100	-57, 257	0.19	34	-91, 159	0.59	-225	-354, -96	0.001	-258	-393, -124	<0.001
Females	152	-466, 769	0.50	78	162, 319	0.49	-61	-301, 178	0.59	-140	-286, 7	0.06
Males	57	-73, 188	0.35	-50	-224, 124	0.55	-316	-457, -174	<0.001	-266	-463, -68	0.01
Peak VO_2, *ml/kg/min*	2.0	-3.3, 7.3	0.43	-0.6	-4.5, 3.3	0.76	-6.1	-9.8, -2.4	0.002	-5.5	-9.5, -1.6	0.007
Females	4.0	-10.1, 18.1	0.48	-0.7	-6.4, 5.1	0.82	-2.7	-8.3, 2.9	0.32	-2.1	-7.0, 2.9	0.40
Males	-1.0	-6.9, 4.8	0.70	-1.2	-6.9, 4.6	0.67	-7.4	-11.7, -3.1	0.001	-6.2	-12.1, -0.3	0.04
Peak VO_2, *% of predicted*	1.3	-11.3, 13,8	0.83	-3.4	-12.2, 5.3	0.44	-10.6	-18.2, -3.0	0.007	-7.2	-15.1, 0.8	0.08
Females	9.5	-24.5, 43.6	0.49	-2.4	-16.3, 11.6	0.73	-7.1	-20.5, 6.2	0.28	-4.8	-16.6, 7.0	0.41
Males	-2.3	-13.3, 8.7	0.65	-2.7	-13.5, 8.1	0.60	-14.4	-22.6, -6.3	0.001	-11.7	-22.7, -0.63	0.04

Abbreviations: Diff: difference; RER: respiratory exchange ratio; Ti/Ttot: Inspiratory time/Total inspiratory and expiratory time ratio; VO_2: oxygen consumption.
Breathing reserve is the difference between maximal voluntary ventilation (FEV_1 x 35) and peak minute ventilation as percentage of maximal voluntary ventilation; 95%CI: 95% confidence interval.
p) Independent sample t-test (equal variance not assumed).
Number of subjects: LVCP: n = 13 (5 females), No LVCP: n=12 (7 females), PDA-surgery: n=25 (12 females), EP-born controls: n=30 (17 females), term-born controls: n=36 (13 females). Mean difference = the first mentioned group minus the last-mentioned group.

Supplementary figure 1 Self-reported physical activity among the EP-born participants who underwent neonatal PDA surgery with and without left vocal cord paralysis (LVCP)

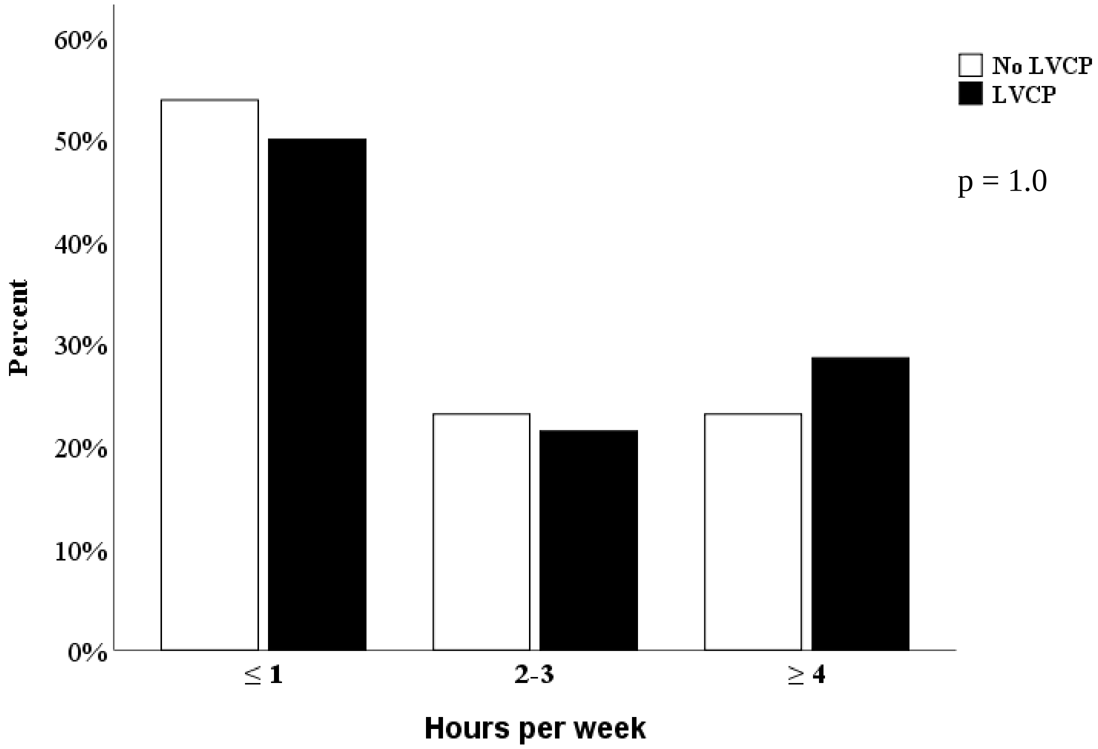

Answer to the self-reported question *"How many hours per week do you attend sports, exercise, or exert yourself so much that you get out of breath and/or sweat?"*

Response rate: No LVCP: n = 13/13, LVCP: n = 14/14,

p) Fisher's exact test

Supplementary figure 2 Comparison of visually assessed laryngeal obstruction during exercise (modified CLE score) according to self-reported breathing symptoms in extremely preterm born adults that underwent neonatal patent ductus arteriosus surgery

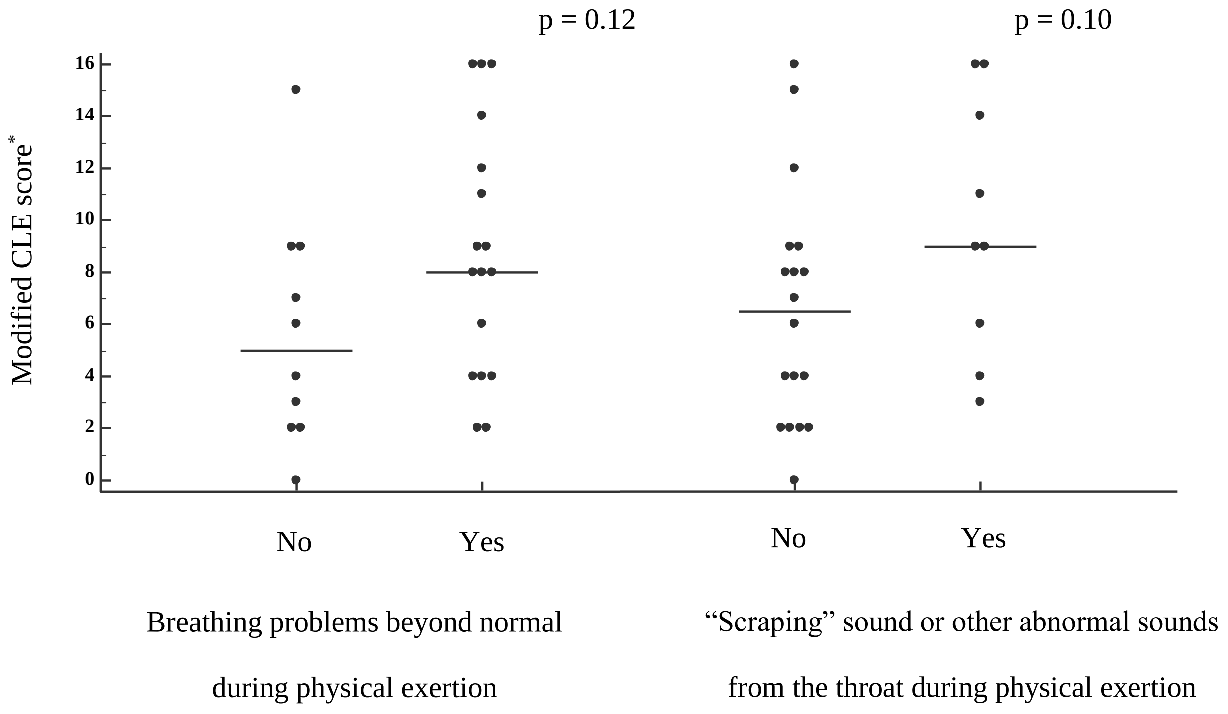

*) Higher modified CLE score indicate more laryngeal obstruction

Median values are indicated by vertical lines

p) Mann-Whitney U test
